# Supplementary material for: Abnormal brain state distribution and network connectivity in a SYNGAP1 rat model
Source: Brain Commun. 2022 Oct 15;4(6):fcac263. doi: 10.1093/braincomms/fcac263 (PMC9638780; doi:10.1093/braincomms/fcac263)
Supplement: fcac263_Supplementary_Data [file fcac263_supplementary_data.pdf]

Supplementary Material

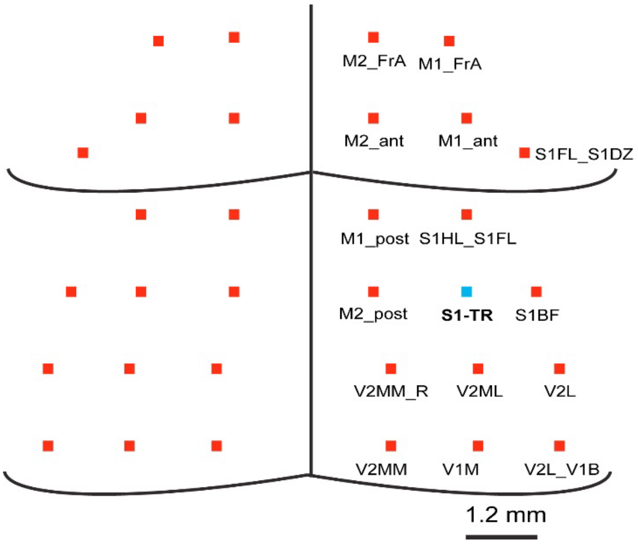

Supplementary Figure 1. Diagram of approximate electrode location and abbreviations of approximate underlying cortical areas.
Coordinates were based on approximate locations from (Paxinos and Watson, 1998)

V2L_V1B: Secondary visual cortex lateral area – Primary visual cortex binocular area
V1M: Primary visual cortex monocular area
V2MM: Secondary visual cortex mediomedial area
V2L: Secondary visual lateral area
V2ML: Secondary visual mediolateral area
V2MM_R: Secondary visual cortex mediomedial area - retrosplenial agranular area
S1DZ_S1BF: Primary somatosensory cortex barrel Field
S1Tr: Primary somatosensory cortex trunk area
M2_post: Posterior secondary motor cortex
S1HL_S1FL: Primary somatosensory cortex hindlimb region – forelimb region
M1_post: Posterior primary motor cortex
S1FL_S1DZ: Primary somatosensory cortex forelimb region - disgranular region
M1_ant: Anterior primary motor cortex
M2_ant: Anterior secondary motor cortex
M1_FrA: Primary motor cortex – frontal association cortex
M2_FrA: Secondary motor cortex – frontal association cortex

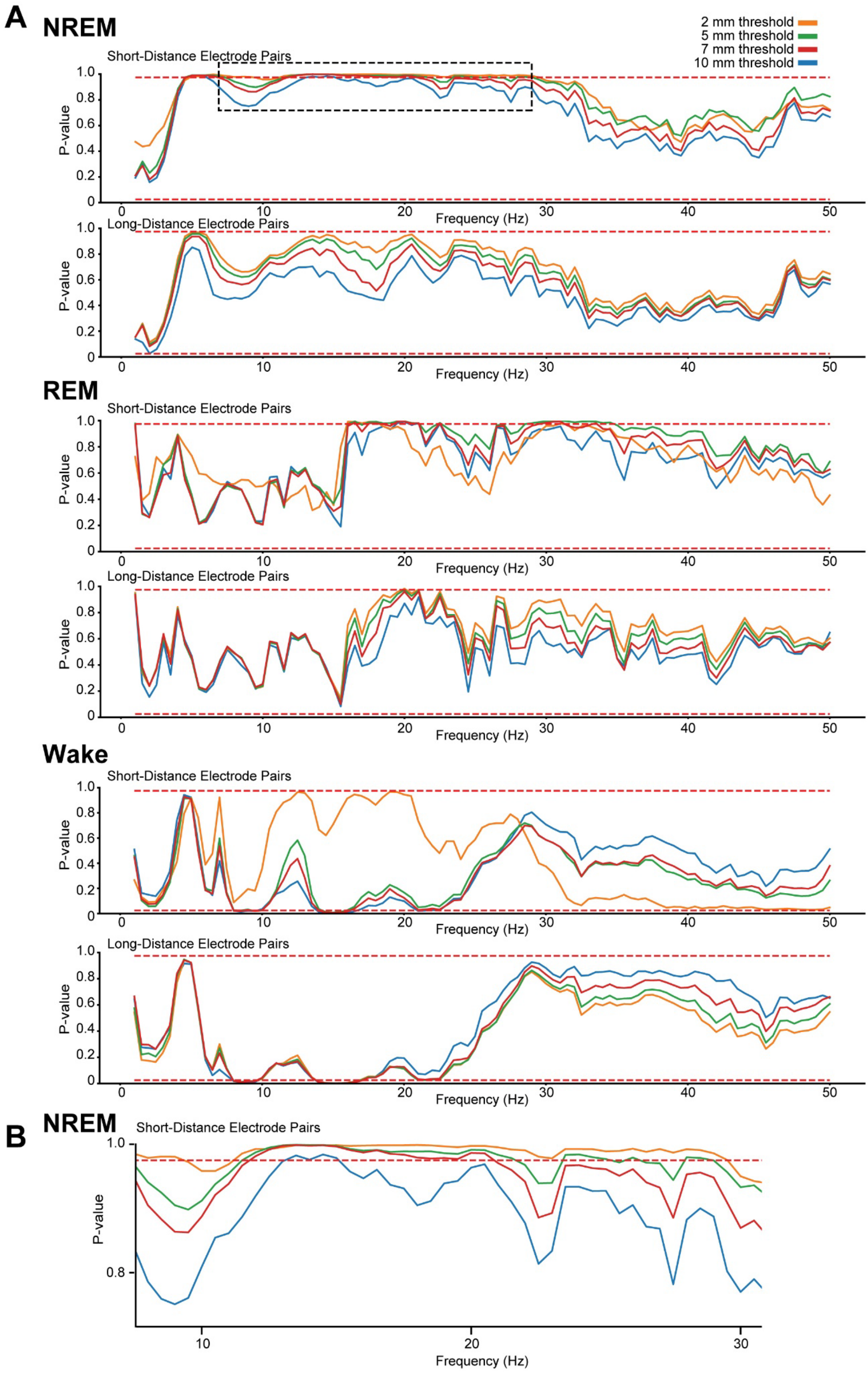

A
NREM
2 mm threshold
5 mm threshold
7 mm threshold
10 mm threshold
Short-Distance Electrode Pairs
Long-Distance Electrode Pairs
REM
Short-Distance Electrode Pairs
Long-Distance Electrode Pairs
Wake
Short-Distance Electrode Pairs
Long-Distance Electrode Pairs
P-value
Frequency (Hz)
B
NREM
Short-Distance Electrode Pairs
P-value
Frequency (Hz)

Supplementary Figure 2. Plots of p-values at multiple distance thresholds. (A) P-values of differences at individual frequencies comparing imaginary coherence between *Syngap*$^{+/\Delta\text{-}GAP}$ and *Syngap*$^{+/+}$ rats for cluster-based nonparametric tests for short and long distance electrodes during NREM, REM and wake. Dotted red lines indicate two-sided p-vale thresholds of ≥ 0.975 and ≤ 0.025 corresponding to significantly different thresholds equivalent to p ≤ 0.05. Note: The distance threshold determines how electrode combinations are grouped as short or long distance combinations. At thresholds of 2, 5, 7 and 10 mm there were 20, 170, 284 and 432 electrodes in the short distance groups and 476, 326, 212 and 64 electrodes in the long distance groups respectively. (B) Expanded p-value and frequency plot of area within the dotted black rectangle in (A). Note: A long cluster of significant frequencies was found during NREM between 11.5 and 29.5 Hz in electrodes ≤ 2mm indicating a decrease in Z' imaginary coherence in *Syngap*$^{+/\Delta\text{-}GAP}$ rats. The longest consecutively significant frequencies clusters are present amongst short-distance electrodes ≤ 2mm apart.

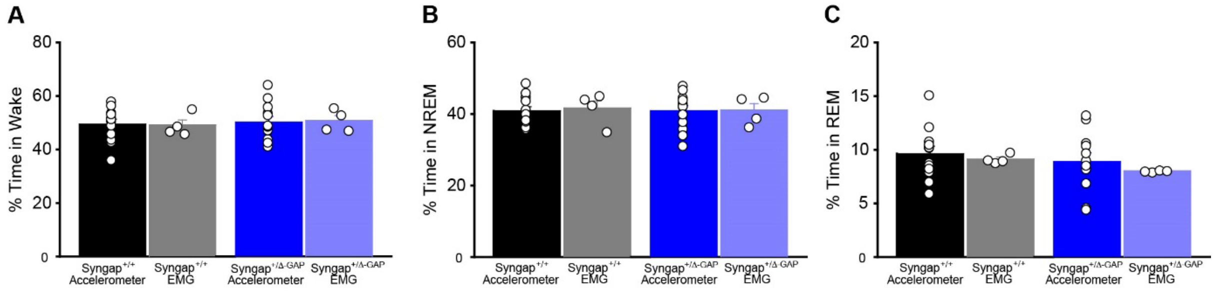

Supplementary Figure 3. Validation of visual scoring obtained through EEG with head accelerometer against standard EEG+EMG. (A) Comparison of percentage of time in Wake, (B) NREM and (C) REM, obtained by visual scoring with accelerometer (Fig. 1B) with 4 *Syngap*$^{+/\Delta\text{-}GAP}$ and 4 *Syngap*$^{+/+}$ rats recorded with EEG+EMG (see methods). Two-way ANOVA for independent measures did not reveal significant differences for factor Genotype, factor Method, or for the interaction between Genotype x Method. Comparison between genotypes for the results obtained using the EMG+EEG method did not show significant differences for percentage in Wake (2-tailed t-test: DF = 6, T= 0.581, p=0.583; *Syngap*$^{+/+}$: 49.15 ± 1.82; *Syngap*$^{+/\Delta\text{-}GAP}$: 49.15 ± 1.82), NREM (unpaired 2-tailed t-test: DF = 6, T= 0.193, p=0.853; *Syngap*$^{+/+}$: 41.7 ± 1.98; *Syngap*$^{+/\Delta\text{-}GAP}$: 41.11 ± 1.78), or REM (unpaired 2-tailed t-test: DF = 6, T= 0.174, p=0.868; *Syngap*$^{+/+}$: 9.14 ± 0.19; *Syngap*$^{+/\Delta\text{-}GAP}$: 8.03 ± 0.03), thus confirming the trend found using accelerometer. Bars indicate mean values (mean ± SEM). Points correspond to values from individual rats.

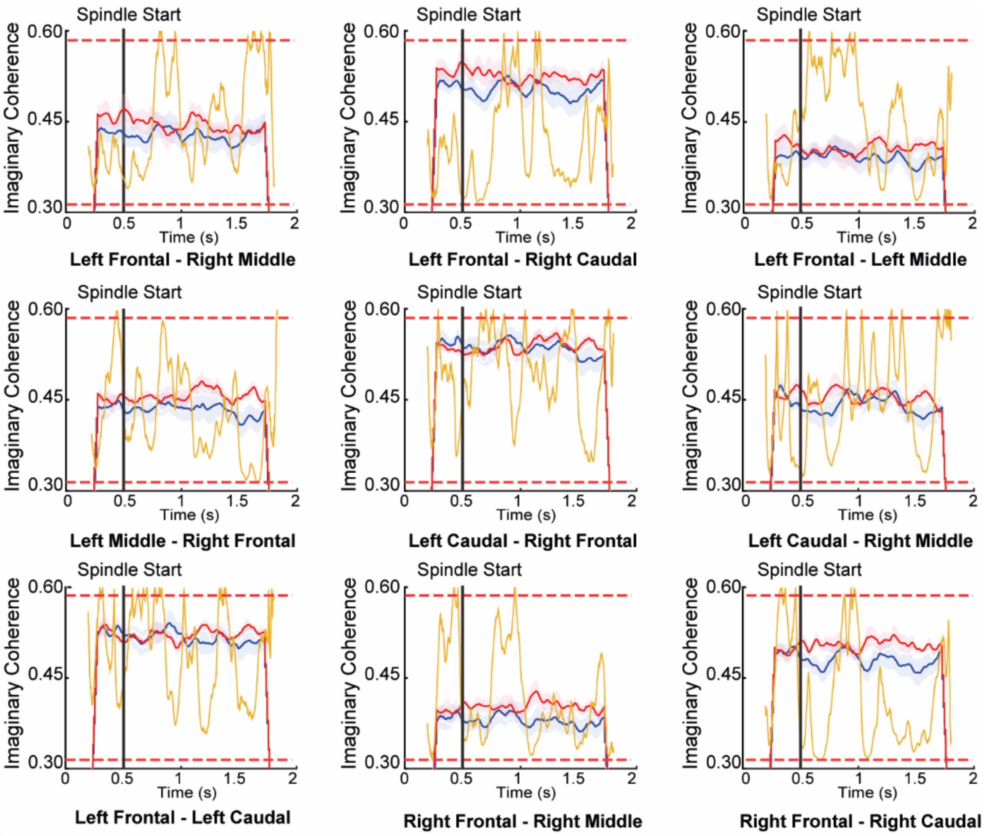

Supplementary Figure 4. Plots comparing average imaginary coherence preceding and during sleep spindles between *Syngap*$^{+/\Delta\text{-}GAP}$ and *Syngap*$^{+/+}$ rats. P-values for cluster-based nonparametric test displayed in yellow with dotted red lines indicating two-sided p-value thresholds of ≥ 0.975 and ≤ 0.025 corresponding to significantly different thresholds equivalent to $p \leq 0.05$, two sided. Note: Comparison schematic and other comparisons are displayed in main text Fig. 7.

Supplementary Table 1. Electrode pairs during sleep spindles with significantly decreased dynamic imaginary coherence. All 496 electrode pairs were compared across animals with a two-sample t-test. The 45 significant pairs are listed below with corresponding p-values.

Electrode Pairs		p-values
V2L_V1B_RIGHT	S1Tr_LEFT	0.031
V2L_RIGHT	S1Tr_LEFT	0.033
V2ML_RIGHT	S1Tr_LEFT	0.031
V2MM_RSA_RIGHT	S1Tr_LEFT	0.049
M2_ant_RIGHT	S1DZ_S1BF_LEFT	0.042
V2L_V1B_RIGHT	S1DZ_S1BF_LEFT	0.003
V2L_RIGHT	S1DZ_S1BF_LEFT	0.007
V2ML_RIGHT	S1DZ_S1BF_LEFT	0.050
S1DZ_S1BF_RIGHT	S1DZ_S1BF_LEFT	0.035
V2MM_RIGHT	V1M_LEFT	0.035
V2L_V1B_RIGHT	M2_post_LEFT	0.028
V2L_RIGHT	M2_post_LEFT	0.038
V2MM_RSA_RIGHT	M2_post_LEFT	0.028
V2L_V1B_RIGHT	S1HL_S1FL_LEFT	0.050
V2L_RIGHT	S1HL_S1FL_LEFT	0.043
V2L_V1B_RIGHT	M1_post_LEFT	0.017
V2L_RIGHT	M1_post_LEFT	0.017
V2ML_RIGHT	M1_post_LEFT	0.031
V2MM_RSA_RIGHT	M1_post_LEFT	0.040
S1DZ_S1BF_RIGHT	M1_post_LEFT	0.029
V2L_RIGHT	S1FL_S1DZ_LEFT	0.049
M1_FrA_RIGHT	M1_ant_LEFT	0.033
V2L_V1B_RIGHT	M1_ant_LEFT	0.036
V2L_RIGHT	M1_ant_LEFT	0.041
M2_ant_RIGHT	M2_ant_LEFT	0.029
V2L_V1B_RIGHT	M2_ant_LEFT	0.021
V2L_RIGHT	M2_ant_LEFT	0.022
S1DZ_S1BF_RIGHT	M2_ant_LEFT	0.037
M1_FrA_RIGHT	M2_FrA_RIGHT	0.046
V2L_V1B_RIGHT	M2_ant_RIGHT	0.018
V2L_RIGHT	M2_ant_RIGHT	0.020
S1DZ_S1BF_RIGHT	M2_ant_RIGHT	0.020
V2L_RIGHT	M1_ant_RIGHT	0.026
S1DZ_S1BF_RIGHT	M1_ant_RIGHT	0.042
V2L_RIGHT	S1FL_S1DZ_RIGHT	0.025
S1DZ_S1BF_RIGHT	S1FL_S1DZ_RIGHT	0.044
V2L_V1B_RIGHT	M1_post_RIGHT	0.015
V2L_RIGHT	M1_post_RIGHT	0.008
V2ML_RIGHT	M1_post_RIGHT	0.037
S1DZ_S1BF_RIGHT	M1_post_RIGHT	0.004
V2L_RIGHT	V2L_V1B_RIGHT	0.002
V2ML_RIGHT	V2L_V1B_RIGHT	0.038
S1DZ_S1BF_RIGHT	V2L_V1B_RIGHT	0.008
S1Tr_RIGHT	V2L_V1B_RIGHT	0.023
S1Tr_RIGHT	V2L_RIGHT	0.020
